# The Semantics of the Preposition “^
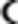
^*alā*” in the Quran: A Conceptual Metaphor Perspective

**DOI:** 10.3389/fpsyg.2022.788582

**Published:** 2022-07-20

**Authors:** Khan Sardaraz, Radzuwan Ab Rashid, Aasia Nusrat

**Affiliations:** ^1^Office of Registrar, University of Science and Technology Bannu, Bannu, Pakistan; ^2^Faculty of Languages and Communication, Universiti Sultan Zainal Abidin, Kuala Terengganu, Malaysia; ^3^Department of Humanities, COMSATS University Islamabad, Lahore, Pakistan

**Keywords:** preposition, conceptual metaphor (CM), metaphor, conceptual system, sensorimotor neural structure

## Abstract

Traditional syntactic approaches do not offer a plausible explanation regarding the use of the Arabic preposition “^
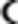
^*alā*” with abstract nouns or states. This article adopts a corpus-based approach to investigate the semantic classification of the preposition “^
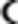
^*alā*” in the Quran from a cognitive linguistic perspective. Conceptual metaphor theory (hereafter CMT) was employed to find out conceptual metaphors (hereafter CM) in the data retrieved from the Quran with the help of search Quran software. CMT holds that human sensorimotor neural structures help store spatial relationships, which are then used to map the abstract concepts in language and thought, and that prepositions are the products of human sensorimotor neural structures. This paper found nine key CM themes behind the usage of prepositions in the data. Contact and support schemas were at the heart of the literal and metaphorical use of the spatial preposition “^
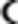
^*alā*” in the Quran. However, it was also found that language generation and comprehension involve the role of multimodal perceptual schemas and linguistic knowledge rather than the unilinear process of one CM. This paper suggests further research into spatial relations across languages to explore the cross-cultural implications of image schemas.

## Introduction

In a syntactic model of prepositions, the preposition “^
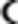
^*alā*” has extensively been discussed in previous literature. Syntactically, “^
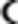
^*alā*” is a separable preposition and may be a link between a verb and an object or serve as a noun. As a preposition, “^
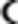
^*alā*” has been grouped with prepositions, “^
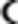
^*an*”, “*munz*”, “*muz*”, and “*kāf* ”, which may either precede a noun or a word and turn the noun into a genitive case (Al-Sayyuti, 1985). The particle “^
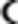
^*alā”* may occur in the accusative case as a noun. Aldwayan (2013) holds that the preposition “^
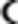
^*alā*” is a place relator preposition that links a FIGURE to a particular space within the ground geometry and requires a DP complement (an object in length). “^
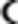
^*alā*” also carries semantic functions. Semantically, this preposition has sparked an elaborate discussion in traditional grammar literature. The famous book on Arabic grammar, “*hidayat un Nahw*”, claims that the primary sense of the preposition “^
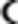
^*alā*” is “*isthe'lā*”-“*elevation*” may also be used as a noun (Andulusi, 2008). Al-Sayyuti (1982) holds that this preposition carries the basic sense of “*above*”, “*on*”, and “*upon*”, but it may also be used for other purposes, such as “*ma*^
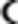
^*a*”-”*with*”, “*min*”-”*from*”, “*li*”-”*for the reason of* ”, “*fi*”“-”*in*” and “*ba*”-“*because of* ”. In other words, the preposition “^
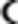
^*alā*” may attain different semantic arguments of different prepositions in different contexts.

Haywood and Nahmad (1962) and Badawi et al. (2013) have also carried out the semantic classification of Arabic prepositions. According to Haywood and Nahmad (1962), the preposition “^
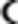
^*alā*” may be used in different senses, such as “*on*”, “*over*”, and “*against*”, the meaning of notice, sense of hostility, and sense of burden, debt, and obligation. Similarly, Badawi et al. (2013) argue that a preposition may have a basic, extended, or figurative sense. In its primary sense, “^
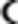
^*alā*” as a preposition may have the semantic value of “*on*”, “*over*”, and “*above*”. However, it may also be used in the sense of obligation, duty, circumstance, state and condition, location, direction, and time, besides its varied usage in compound noun phrases.

The semantic classification of “^
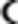
^*alā*” as a preposition encompasses different semantic arguments, which it may take according to the linguistic contexts. However, the received view of prepositions does not orient us to the framework of its various possible meanings or their metaphorical usage. In other words, the use of spatial prepositions with abstract concepts, emotions, and states carries a metaphorical connotation determined by the linguistic context. However, the use of prepositions in a metaphorical sense cannot be logically explained in the received view of prepositions because of the rootless form of prepositions (Sardaraz and Ali, 2017). The metaphoricity and the semantic patterns of prepositions can be explained from a cognitive linguistic perspective, which does not allocate language use to syntactic categories but, rather, human cognition. Cognitive linguistics theories hold that prepositions are generated by perceptual schemas in the human mind, which draw on the human experiences of the relationship of objects in the outside physical world. As outlined below, cognitive-linguistic approaches have already been applied to investigating Arabic prepositions.

Aldwayan (2013) studied Arabic spatial prepositions from a cognitive linguistic perspective. While modern standard Arabic and English share similarities in terms of spatial representation of abstract states, there are differences in the geometrical model of spatial contexts. English tilts toward using a support schema through various spatial prepositions, such as “*on* and *upon*”; Arabic is more tilted toward using the container schema in spatial scenes representation by using the preposition “*fi*”-”*in*”. Both languages use either a container schema or a support schema in conceptualizing states. The support schema is underlying the use of the preposition “^
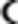
^*alā*” in the Arabic language. The support schema is not merely based on physical experiences of gravity, but it has also sub-schemas of attachment, encirclement, and danglings (Aldwayan, 2013). However, Aldwayan (2013) approach is not corpus based to investigate the metaphorical instances of “^
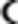
^*alā*” in diverse contexts, which is needed to examine the semantic complexity of the said preposition because meaning depends upon the fusion of linguistic knowledge and conceptual knowledge. Conceptual knowledge consists of cognitive models and conceptual metaphors (Evans, 2006, 2009, 2013). Thus, conceptual metaphor is one of the essential knowledge structures besides linguistic knowledge, which needs due attention in interpreting prepositions. This paper primarily focuses on “^
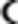
^*alā*” from the CMT perspective besides the role of linguistic knowledge in meaning construction. Sardaraz and Ali (2021), following Evans (2013), call for a more elaborate mechanism that can take into account both linguistic knowledge and conceptual knowledge in interpreting the Quran.

Cognitive semantic approaches offer a better methodology for the semantic classification of Arabic prepositions. Sardaraz and Ali (2017) have investigated the semantics of the preposition “*fi*”-”*in*” in the Quran from a cognitive semantic perspective. They argue that “*fi*”-”*in*” represents the container schema in spatial and non-spatial relations. It has also been used in various novel ways that reflect the importance of linguistic context in meaning construction. Jan (2018) has applied Tyler and Evans (2003) principled polysemy model to the investigation of the prepositions “^
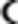
^*alā*” “*on*”, “*over*”, “*above*”, and “*fi*”-“*in*” in modern standard Arabic and claims that “^
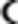
^*alā*” has the immediate sense of locating a target contacting its base or being higher than the base in the space. This immediate sense provides the basis for all the metaphorical senses, spatial or non-spatial. Broadly, the secondary sense of the preposition “^
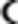
^*alā*” can be categorized as senses that are not clustered, elevation senses and senses of nearness. Jan's study has contributed a lot to the functional semantics of the preposition “^
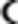
^*alā*” in modern standard Arabic; yet it has not elaborated the role of CM in the usage of spatial prepositions in metaphorical sentences, as is the case with the following sentence:

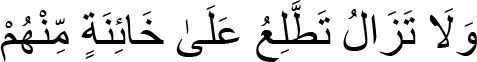

  “*walā tazālu taṭṭali*^
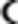
^*u* ^
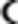
^*alā khāinatin min'hum*”
And not will you cease to discover of treachery from them (Quran 5:13)

The noun “*khāinatin*”-”*treachery*” is derived from the root “*khā wāw nūn*”. The root embodies the concept of lessening or contracting something. The lexical unit “*khāinatin*” means treachery or deceit. However, deception cannot be discovered in a concrete, substantive sense, and, therefore, it is used metaphorically. The lexical unit “*khāinatin*” combined with the preposition “^
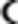
^*alā*” links to a physical object that can be discovered. Hence, “^
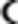
^*alā khāinatin*”-”on *deceit*” or “*of treachery*” is represented as a physical object that can be found or observed. While this example illustrates an extension of the primary meaning of “^
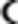
^*alā*”, this seems too simplistic on closer examination. The principled polysemy model offers better insights into the meaning of prepositions in diverse contexts but leaves out the CM. If CM is left out, any study may miss the bulk of instances of metaphorical usage of a preposition. Moreover, if the principled polysemy model offers a better approach to the semantic network of a particular preposition on the basis of its proto sense and extended senses, CM has also proved a more viable model to the categorization of spatial prepositions in metaphorical sentences on the basis of our experiential relationship with the outside world (see Sardaraz and Ali, 2017, 2020; Sardaraz et al., 2019). Therefore, the preposition “^
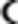
^*alā*” needs to be investigated from the CM perspective to reveal one of the essential knowledge structures as held by Evans (2013). In the above sentence, the lexical items “^
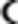
^*alā khāinatin*”-“on *deceit*” instantiate the CM of AN ABSTRACT CONCEPT IS A PHYSICAL OBJECT.

Sardaraz et al. (2019) have investigated the preposition “*min*” in the Quran from a cognitive linguistic perspective. They argue that the source-path-goal schema is behind the usage of the preposition “*min*” in Arabic. However, they hold that the semantic argument of the preposition is strongly dependent upon the situational context, and its meaning may vary from place to place. This study also recommended further research into the same or other Arabic prepositions from a cognitive linguistic perspective. Thus, the available literature reveals that the Arabic prepositions provide a vibrant field for research, and this paper investigates the CMs involved in using the preposition “^
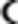
^*alā*” in the Quran.

Each situational context adds distinct semantic value to the preposition. In other words, the meaning depends on CM and the immediate linguistic context (Evans, 2013). According to Evans (2013), language processing and comprehension involve front stage and backstage cognition. CM, no doubt, structures the primary cognitive models, but a lexical concept in the immediate linguistic context may achieve access to secondary cognitive models if there is a clash in primary cognitive models for contextual meaning. For example, in the sentence, *he is in pain*, the container schema is activated, but the *pain* is not a container in the geometric sense. Clash arises in primary cognitive models, and the conflict is resolved once it gets access to state sense, drawing upon the experiential knowledge of States as Containers. The semantic values of the lexical concept *pain* would take is not enclosure, but the affecting condition. It can be argued that language comprehension is a bidirectional traffic mechanism involving both language and conceptual schemas (Sardaraz and Naeem, 2019).

**Figure 1:**
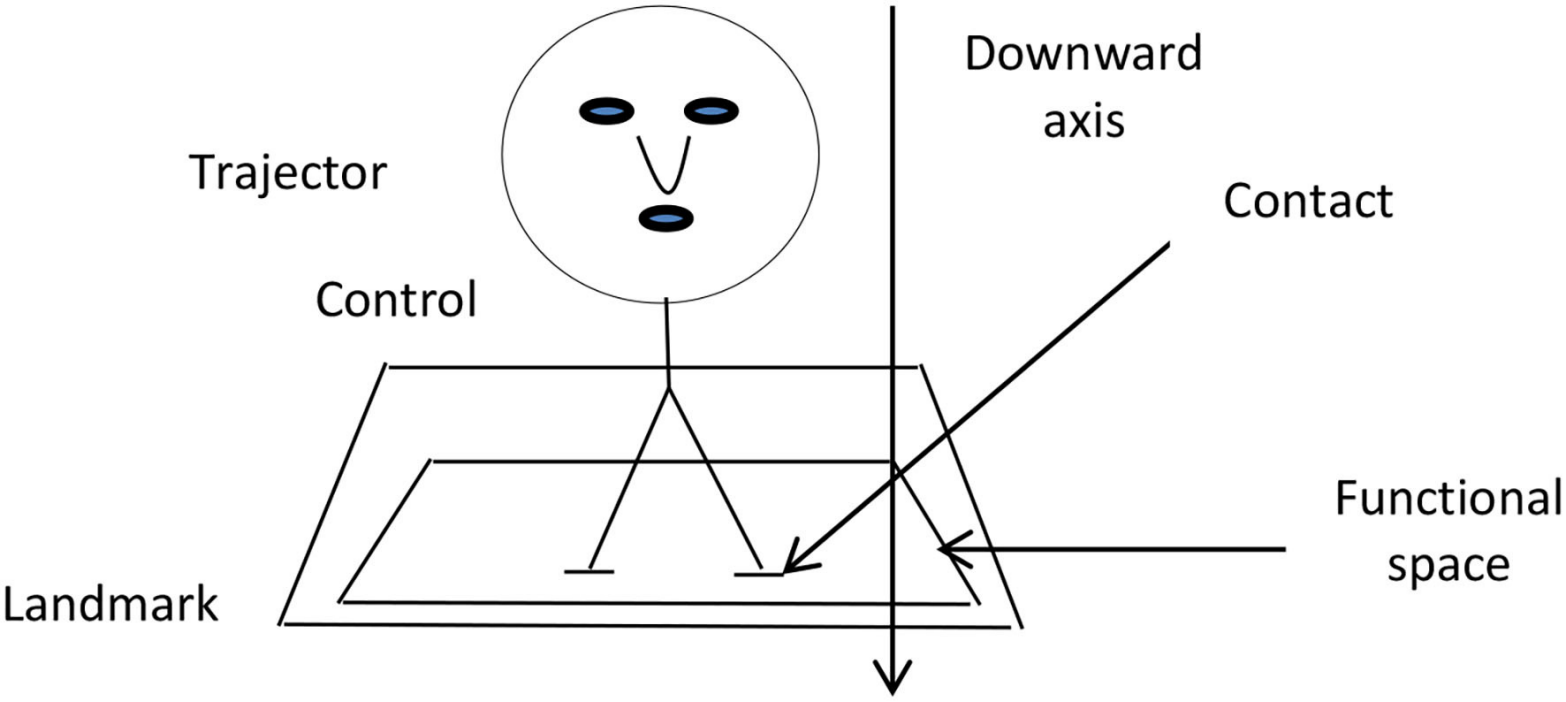
Support and contact schema for*alā*.

In a CM, the target concept is structured in light of the source concept but holds that the metaphor is the product of the conceptual system, manifested in language. It has no abstract linguistic value *per se*, as claimed in the rhetorical tradition. For example, the sentence:

2. *he is in pain*

would be processed literally in the rhetorical tradition. Still, from the CM perspective, it is said to be generated by the CM STATES ARE LOCATIONS, as the preposition “*in*” gives locational status to the emotions of “*pain*”. As referred to earlier, prepositions (postpositions) in language are generated by different conceptual schemas cross-linguistically, such as container schema, support, and contact schemas or source-path-goal schemas, orientation schemas, visual schemas etc. (see Johnson, 1987; Sinha and Thorseng, 1995; Lakoff and Johnson, 1999), and Jan (2018) holds that “*alā*” is generated by a support schema. The conceptual schema for support has been represented in literature (Ferrando, 1999) diagrammatically as in Figure 1.

Figure 1 presents the relationship of objects in space involving support, contact, and gravity between an object and the functional space in which it is located. It represents the trajector (TR) exerting a downward force on the landmark (LM), having actual contact with the functional space of the LM. The LM supports the TR from below in the available space. The TR has contact with the LM and the support of the LM. This is a diagrammatical representation of a support schema in the conceptual system that draws on our experiences in the outside physical world and our knowledge of the relationships between objects in physical space. For instance, the sentence, “*The cup is on the table*”, shows that the relationship between the “*cup*” (TR) and the “*table*” (LM) is one of the supports given by the table to the cup and reflects the contact between the “*cup*” and the “*table*”. The preposition “*on*” is used here in its primary meaning of support. However, linguistic data also reveal that there may be no contact or support, as is the case with “*handle on pan*”, where there is no real support but a sense of attachment between the “*handle*” and the “*pan*” (Aldwayan, 2013). In Arabic, a support schema is used in the following clause, encoding the primary meaning of the preposition “^
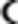
^*alā*”,

3. 
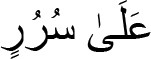
“^
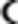
^*alā sururin*”
“on thrones”

The same support schema has been used in metaphorical usage of this preposition with many nouns encoding emotional states, circumstances, and other abstract concepts, such as falsehood, piety, curse, importance, and power. Consider the following sentence:

4. 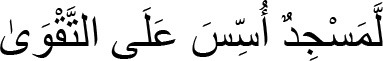
“*lamasjidun ussisa* ^
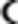
^*alā taqwā*”
“A masjid founded on righteousness” (Quran 9: 108)

The above sentence uses the preposition “^
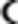
^*alā*” with the abstract concept of righteousness, defining righteousness as a physical object or material. In other words, the spatial preposition “^
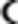
^*alā*” defines the abstract concept “*taqwā*”-“*righteousness*” in spatial, physical terms. This clause is generated by the CM ABSTRACT CONCEPTS ARE PHYSICAL OBJECTS/THINGS. This suggests that CM is a key knowledge structure of the semantic structure of language (Evans, 2009). Hence, this paper investigates the correlations between experiential gestalts and the abstract target domain, which will contribute to the research on metaphorical use of Arabic prepositions in general and “^
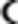
^*alā*” in particular.

## Research Methodology

This paper used Search Quran software, Islam 360, developed by Zahid Hussain (2015) for data collection. This is a comprehensive search engine that not only facilitates in data collection on an input of a lexical entry (Arabic word) for retrieving verses containing that particular lexical item in whole of Quran but also in ontological search on various subjects. The screenshot at **Figure 3** shows the operation of this software.

In other words, both the lexical forms are to be searched separately, and the search gives words and a verse count of each form separately. This search engine has also been tested in previous research (please see Sardaraz and Ali, 2017; Sardaraz et al., 2019). According to Naeem et al. (2019), the worth of this application in knowledge of the Quran is immense. This search engine functions on lexeme-based search by entry of a particular lexeme in the search tab, and it brings all the verses having that lexical entry. For example, 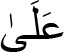 and 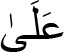 are two lexical forms; the former stands alone in a sentence such as “^
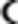
^*alā ṣirāṭin*-on path”, while the latter needs a madda such as “^
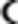
^*alā l-lahi*-upon Allah”.

The search of these two lexical forms helped in retrieving 667 verses. The corpus was manually read, and it was found that 70 verses were repeated either because of the presence of both forms of the preposition or because of more than one occurrence of the preposition in the same verse. The repeated verses were removed. After data distillation, the final corpus for analysis consisted of 597 verses of the Quran. After initial data retrieval, data deconstruction was carried out, and “^
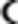
^*alā*” was examined in each particular context, keeping in view any syntactically genitive noun and preceding a noun or a verb, such as “*naṭba*^
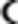
^*u* ^
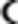
^*alā qulubi*-We seal (on) hearts” (Quran 10:74) where the noun “*qulubi*” is in the genitive case. The whole corpus was broken down into purposeful lexical units with verb/noun + preposition + noun structure. Considering the large data set, pronouns following “^
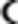
^*alā*” were mostly avoided to concentrate on nouns. The codes were assigned to nouns derived from a single lexeme and placed together in a single set. The coding process helped us in the initial classification of data, which was syntactic. The text”s translation and transliteration were taken from the Noble Quran website (https://corpus.quran.com/wordbyword.jsp).

Each lexical unit was examined in its immediate linguistic context for its metaphorical implications. This process involved reading and determining the meaning of the lexical item in order to define its metaphoricity based on the metaphor identification procedure (Group, 2007). Consider the following clause:

5. 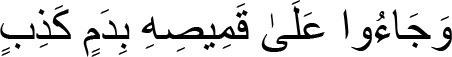
”*wajāū* ^
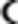
^*alā qamīṣihi bidamin kadhibin*”
“And they brought upon his shirt false blood” (Quran 12:18)

**Figure 2:**
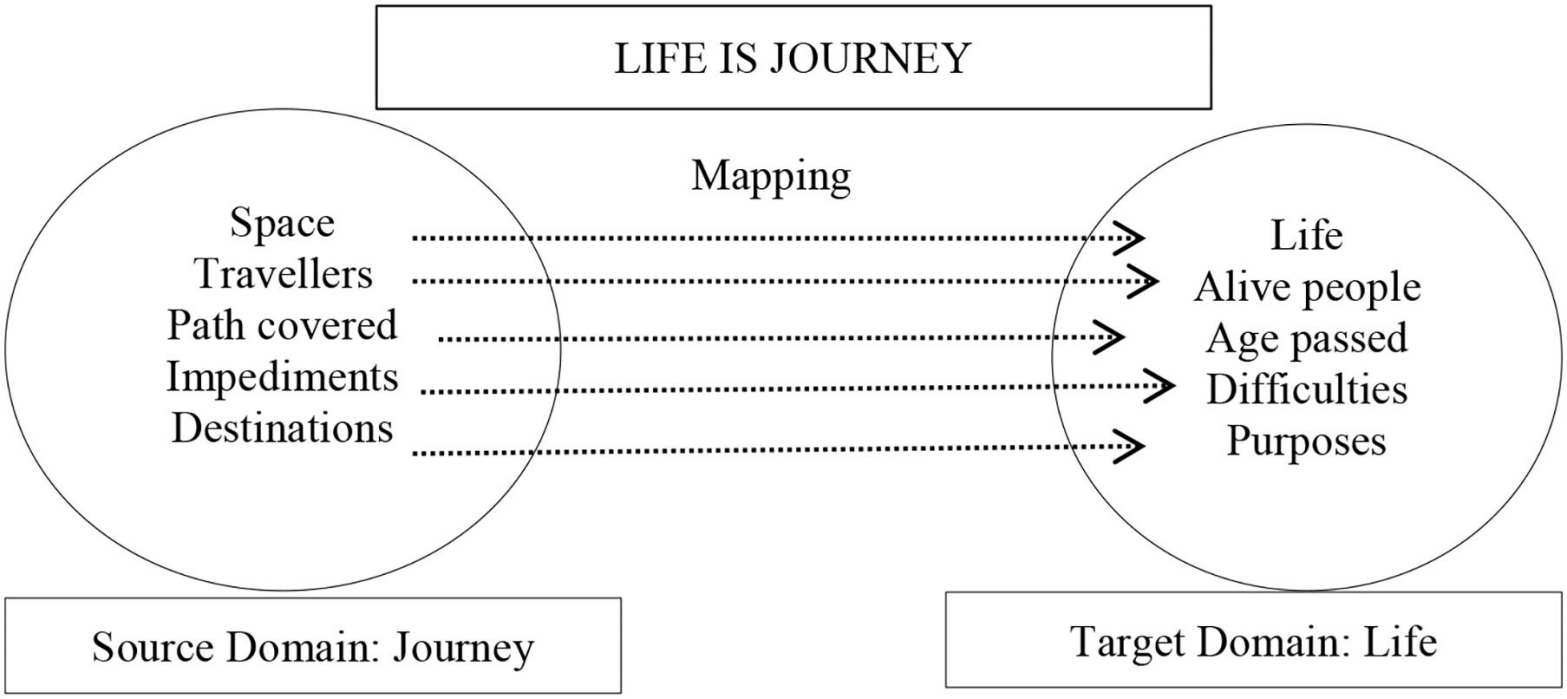
Partial representation of LIFE IS JOURNEY.

In (5), the lexical unit, “^
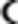
^*alā qamiṣihi*” “*on his shirt*” specifically uses explicitly the preposition “^
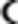
^*alā*” in a spatial geometrical sense because it configures the blood precisely in contact with the shirt, and it involves a support schema. Spatial usage of the preposition “^
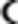
^*alā*” in a strict geometric sense was classed together, while metaphorical lexical units were classified according to CMT (Lakoff and Johnson, 1980; Kövecses, 2002). CMT holds that metaphor is a cross-domain mapping in our conceptual system, wherein an abstract domain is structured in experiential gestalt as LIFE IS JOURNEY, given in Figure 2 (Sardaraz and Nusrat, 2019).

**Figure 3:**
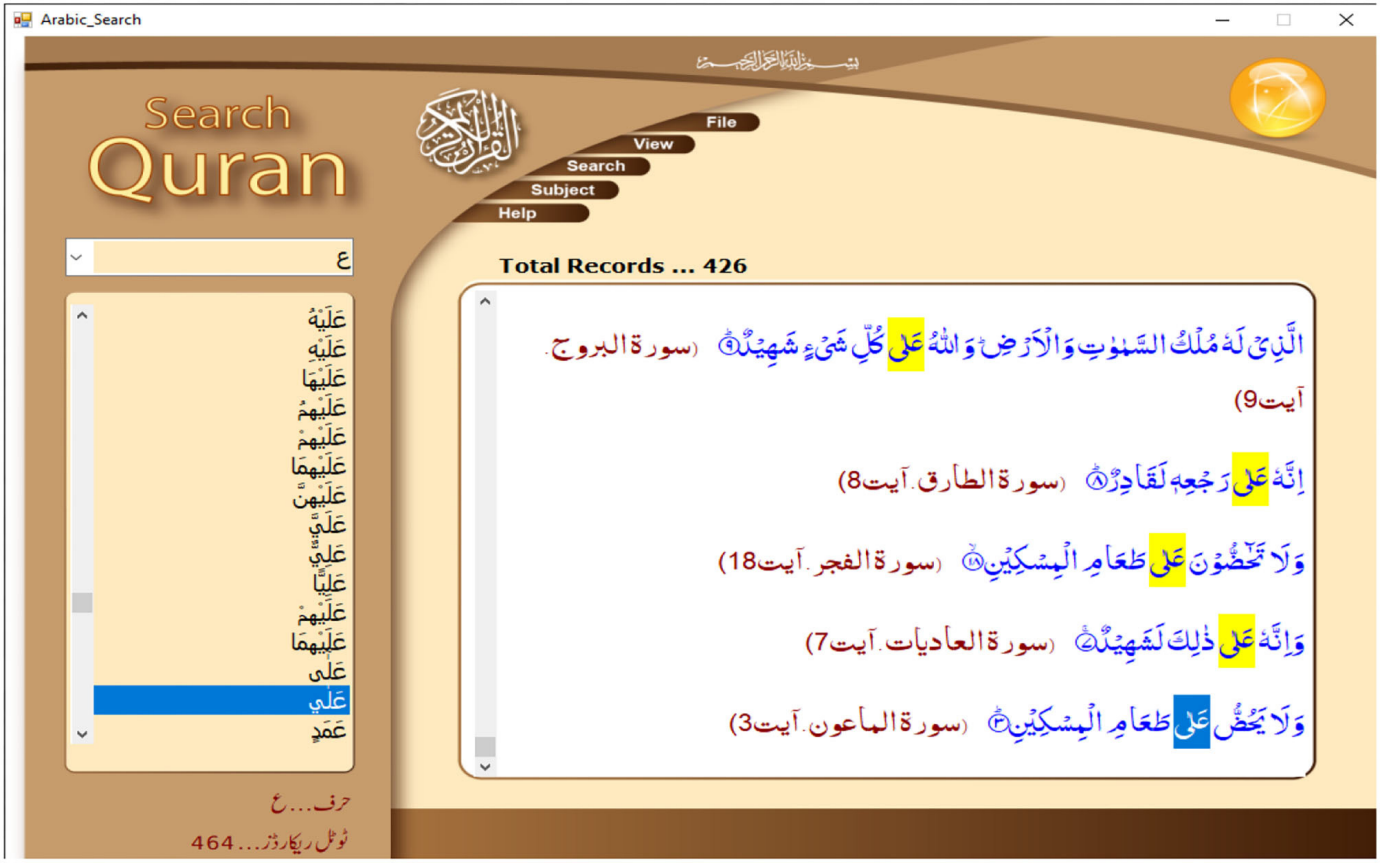
Mechanism of lexical search in search Quran software. Reproduced with permission.

Each metaphorical clause was categorized under one CM theme. For example, duties and responsibilities were classified as a category on the source domain of burden. Within the cognitive linguistic framework, duties and responsibilities are mapped as burden (Kövecses, 2002). The preposition “^
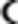
^*alā*” is mainly responsible for the spatial orientation of abstract concepts in terms of human experiences in the physical world. The refined corpus was analyzed through CMT, as given below.

### Data Analysis

The analysis of the data reveals that the preposition “^
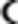
^*alā*” has extensively been used in the Quran to configure the spatial relations in language. However, the data also illustrated many CMs, such as duties are burdens, life is a journey, important is up, power is up, abstract concepts are things, personification, and the metonymic relationship between the heart and the mind through the container schema. Moreover, the preposition has also been used in novel and irregular ways, which the symmetrical and regular patterns of CMT cannot explain. Each major CM found in the data is analyzed below.

### Spatial Sense

The preposition “^
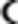
^*alā*” is extensively used with spatial sense, denoting the relationship between physical objects in terms of support and contact. Eighty instances of this usage were found in the data in connection with 49 different nouns, revealing the spatial geometric meaning associated with the preposition “^
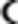
^*alā*”. These instances show support and contact schemas, denoting relationship between physical objects. Some instances are analyzed below.

6. 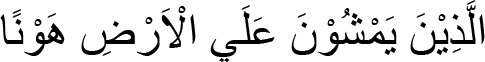
“*alladhīna yamshūna* ^
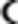
^*alā l-arḍi hawnan*”
“those who walk on the earth in humbleness” (Quran 25:63)

7. 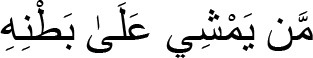
“*man yamshī* ^
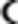
^*alā baṭnihi*”
“who walks on its belly” (Quran 24:45)

8. 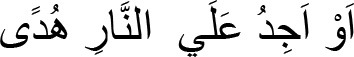
“*aw ajidu* ^
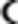
^*alā l-nāri hudan*”
“or I will find at the fire guidance” (Quran 20:10)

9. 
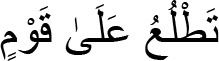
“*taṭlu*^
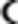
^*u* ^
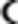
^*alā qawmin*”
“rising on a community” (Quran 18:90)

Spatial relations are illustrated in the above examples. In (6) and (7), the relationship is one of physical support and contact, but with different meanings. In (6), the support is provided by the earth, while, in (7), a part of the body (the belly) supports the rest of the body in walking. In the (8), there is no physical contact between fire and Moses; it conveys the sense of proximity to show the close proximity of the fire in the fireplace and Moses. In (9), the word *taṭlu*^
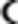
^*u-rising* refers to the sun rising in the morning. There is no physical contact or support between the sun and the community, but it is more of covering sense, involving light falling upon the community from above.

In the following examples, the sense changes according to the context.

10. 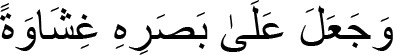
“*waja*^
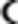
^*alā* ^
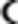
^*alā baṣarihi ghishāwatan*”
“and put over his vision a veil” (Quran 45:23)

11. 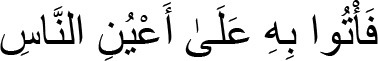
“*fatu bihi* ^
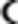
^*alā a*^
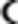
^*yuni l-nāsi*”
“Then bring him before the eyes of the people” (Quran 21:61)

In (10), the sense is that of covering over the vision due to which one cannot understand the truth, and, in (11), the concept of examining is implied. Contextually, both points to the CM of *KNOWING IS SEEING* because both involve mapping between the source domain, seeing and target domains, knowing. However, in (10), there is sense of covering, while, in (11), there is a sense of proximity and examining, bringing someone near the people to look at him or her and examine his or her identity. This shows that, even in the spatial configuration of objects or persons in space, the preposition may indicate a range of semantic concepts.

Spatial relations can acquire metaphorical meanings when the preposition “^
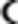
^*alā*” is used with nouns relating to supernatural entities. Thirty-seven instances of this relationship were found with 22 nouns. In the majority of cases examined, a vertical schema was used to denote the spatial relationship between supernatural entities and persons or objects in the physical world. Consider the following.

12. 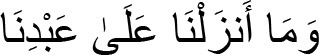
“*wamā anzalnā* ^
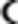
^*alā* ^
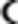
^*abdinā*”
“and in that which We sent down to Our Servant” (Quran 8:41)

13. 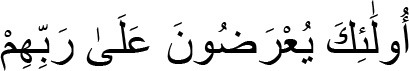
“*ulāika yu*^
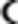
^”*ra*^
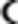
^*una* ^
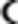
^*alā rabbihim*”
“Those will be presented before their Lord” (Quran 11:18)

14. 
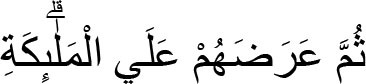
“*thumma* ^
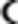
^*araḍahum* ^
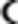
^*alā l-malāikati*”
“Then He showed them to the angels” (Quran 2:31)

(12) is quite metaphorical; Allah is presented in human terms, sending something (revelation) from above to someone, the Messenger. A vertical downward path is used. The contact dimension involves the mind of the Messenger, as the revelation is not a physical object. In (13) and (14), supernatural entities are configured as physical beings; names are mapped as things; people and the names of all things are brought before them through a horizontal pathway, which reflects a sense of proximity. Spatial relationships are described in all three examples above, but, as the entities are supernatural, the relationship described is metaphorical rather than actual. These examples also show that a single utterance may have one or more than one CMs. In (12) and (14) show Great chain of being, and ideas are object metaphors. Thus, language comprehension needs not only involvement of a single CM, but it may depend upon processing of more than one CM.

### Duties/Responsibilities Are Burden Metaphor

The preposition “^
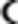
^*alā*” is spatial in nature, but it also gives a spatial character to the circumstances. One of the CMs that give a structure to the state of duty and responsibility is burden. It was found in 26 linguistic expressions as illustrated by the following extracts.

15. 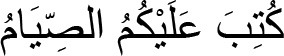
“*kutiba* ^
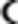
^*alaykumu l-ṣiyāmu*”
“decreed upon you is fasting” (Quran 2:183)

16. 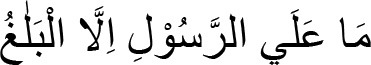
“*mā* ^
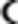
^*alā l-rasūli illā l-balāghu*”
“Not upon the Messenger is except [for] notification” (Quran 5:99)

17. 
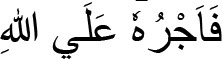
“*fa-ajruhu* ^
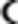
^*alā l-lahi*”
“his reward is [due] from Allah” (Quran 42:40)

In (15) and (16), the duties of fasting and delivering messages are abstract concepts. Injunctions of duties or responsibilities come from high-status persons to lower-status persons, which are adopting the up-down schema. Responsibility is mapped as burden in CMT (Lakoff and Johnson, 1980). Responsibility takes the preposition “^
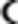
^*alā*” to map it as a burden upon some individual. Hence, “^
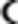
^*alā*” is metaphorically used with semantic argument of “*upon*”, keeping in view the mapping between burden and responsibility. In (17), reward is represented as a burden in the financial term “*ajr*”, and Allah is personified. Thus, any reward for good deeds is mapped as a financial debt to be paid by Allah Himself. Contextually, “*alā*” cannot be interpreted as on or upon when it comes with Allah, but it would mean *from* because of nature of the Being of Allah, higher and greater than everything and that nothing can be burden on Him. In (17) also reflects the involvement of more than a single CM, a great chain of being and responsibility is a burden. If the rewards for good deeds were not represented in mundane language, human beings would not be able to understand.

### Important Is Up Metaphor

The analysis revealed that “^
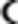
^*alā*” as a spatial preposition is also used to indicate preference or the importance of one object compared to another. This metaphor was found in 27 linguistic expressions. The preposition “^
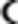
^*alā*” is also used to show the relative importance of one thing, idea or person over another. This relationship between objects is presented in terms of one being over or above others, as in the extracts below.

18. 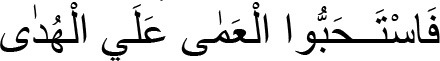
“*fa-is'taḥabbu l-*^
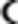
^*amā* ^
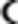
^*alā l-hudā*”
“but they preferred blindness over guidance” (Quran 41:17)

19. 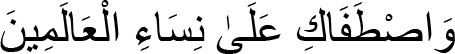
“*wa-iṣ'ṭafāki* ^
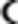
^*alā nisāi l-*^
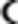
^*ālamina*”
“and chosen you above the women of the worlds” (Quran 3:42)

20. 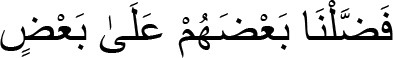
“*faḍḍalnā ba*^
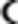
^*ḍahum* ^
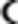
^*alā ba*^
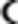
^*ḍin*”
“We have preferred some of them over others” (Quran 2:253)

Extract (18) is highly metaphorical. The preposition “^
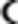
^*alā*” is used with superiority sense, giving spatial configuration to the preference of blindness over guidance, which is an abstract concept configured as a physical object. But the lexical item blindness is in itself metaphorical, generated by ignorance is blindness, the inverse of knowing is seeing metaphor (Vereza and Puente, 2017). In (19) and (20), the significance or importance of one person or persons is presented as being above others by the spatial preposition “^
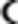
^*alā*”, but, in (19), TR is not in direct influence of LM, while, in (20), LM has direct influence on TR. This also shows that English use *over* and *above* for these senses, Arabic conflates these senses in “^
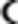
^*alā*”. Thus, the preposition “^
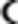
^*alā*” is also used to show the relative importance of two objects, persons, ideas or acts in vertical spatial terms. Moreover, (18) involves the activation of more than one CMs, important is up, and ignorance is blindness.

### Control Is Up

The up schema has also been used to describe control over something through the preposition “^
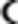
^*alā*”. Control may be over a physical object/entity or an act or activity. Whatever has the power is up or over the object or deed. This schema had sixty instances with 15 different nouns, as in the following examples illustrate.

21. 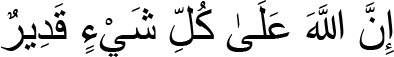
“*inna l-laha* ^
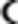
^*alā kulli shayin qadīrun*”
“Allah is on everything All-Powerful.” (Quran 2:20)

22. 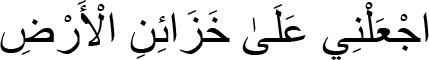
“*ij”* ^
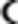
^*alnī* ^
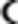
^*alā khazāini l-arḍi*”
“Appoint me upon the treasures of the land” (Quran 12:55)

23. 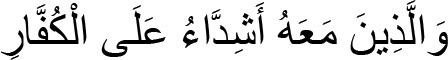
“*wa-alladhīna ma*^
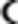
^*ahu ashiddāu* ^
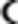
^*alā l-kufāri*”
“and those who are with him are strong over the disbelievers” (Quran 48:29).

In (21), “^
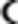
^*alā*” maps all events and physical things in the round as below Allah and which Allah has overall power. Allah is a Supernatural Being, if He had not described himself in human mundane language, the readers of the Quran would be baffled. Similarly, in (22), believers are represented as “above” or “higher than” disbelievers in terms of power and strength. The clause at (23) refers to control over treasures, which are physical objects. In all these examples, “^
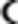
^*alā*” semantically represents “over” or “higher than”, which suggests a downward vertical schema without contact with any FIGURE or the GROUND. Hence, “^
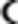
^*alā*” carries the semantic argument of control, as there is both proximity between higher and lower entities and influence of higher upon lower. There is elevation but no contact between objects, except in terms of influence.

## Abstract Concepts are Objects

Abstract concepts, activities, and states are some of the major target domains structured in terms of experiential concepts. Abstract concepts are often presented as physical objects. Seventy instances of this were found in the corpus located with 42 different nouns. Some of the major abstract concepts found in the corpus are mercy/grace, peace, religion, piety, curse, falsehood, and time spatially configured through “^
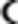
^*alā*”, as in the following phrases.

24. 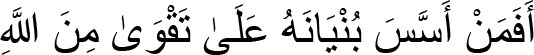
“*afaman assasa bun'yānahu* ^
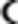
^*alā taqwā mina l-lahi*”
“then is one who founded his building on righteousness from Allah” (Quran 9:109).

25. 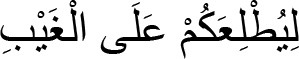
“*liyuṭ”li*^
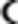
^*akum* ^
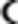
^*alā l-ghaybi*”
“to inform you upon the unseen” (Quran 3:179)

26. 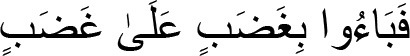
“*fabāū bighaḍabin* ^
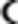
^*alā ghaḍabin*”
“so have they drawn on themselves Wrath upon Wrath” (Quran 2:90).

In the above examples, spatial preposition “*alā*” gives spatial nature to the abstract concepts of “*taqwā*-piety or righteousness”, “*ghayb*-unseen” and “*ghaḍabin*-wrath”, defining them as physical things, as for example, literally, wrath cannot be piled upon the wrath. Hence, “^
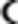
^*alā*” carries the semantic argument of *support* in (24), more sense in (26) and examining sense in (25). Moreover, in (24) and (26), the arrow is in the upward direction, as laying foundation upon and piling upon goes in size in the upward direction, while, in (25), the gaze of the onlooker is on the object and the direction is downward, as if examining something just below our gaze.

However, “^
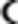
^*alā*” has a different use in the following instances.

27. 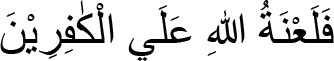
“*fala* ^
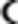
^*natu l-lahi* ^
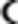
^*alā l-kāfirīna*”
“so the curse of Allah is on the disbelievers” (Quran 2:89).

28. 
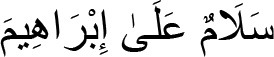
“*salāmun* ^
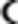
^*alā ib”rāhīma*”
“peace be on Ibrahim” (Quran 37:109).

29. 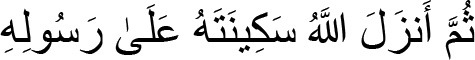
“*thumma anzala l-lahu sakīnatahu* ^
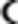
^*alā rasūlihi*”
“then Allah sent down His tranquility on His Messenger” (Quran 9:26).

In (27), (28), and (29), “^
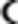
^*alā*” gets the semantic potentials of something from above on someone, defining the abstract concepts “*la*^
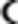
^*natu*-curse”, “*salāmun-*peace”, and “*sakinata*-tranquility” in terms of physical objects. All these concepts are conceived as physical coverings as they encompass the whole person of a human. For example, *la*^
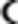
^*natu* cannot be literally interpreted to be on someone, but its metaphorical sense of physical cover can be used with “*alā*”. Thus, the context mediates the meaning, although CM structures these concepts. Also, (27) and (29) reflect that these utterances are processed through more than one CM as besides abstract concepts are objects metaphor, Allah is presented as personified being.

### Acts/Activities Are Things

Activities and acts are mapped as things. According to Lakoff and Johnson (1980), “We use ontological metaphors to comprehend events, actions, activities, and states. Events and actions are conceptualized metaphorically as objects, activities as substances, states as containers.” These conceptualizations were found in 33 instances in the corpus, some of which are analyzed below.

30. 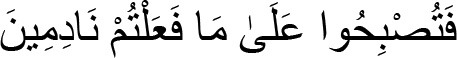
“*fatuṣ'biḥū* ^
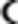
^*alā mā fa* ^
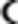
^*altum nādimīna*”
“then you become over what you have done regretful” (Quran 49:6)

31. 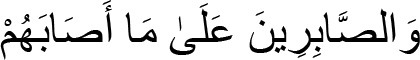
“*wal-ṣābirina* ^
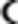
^*alā mā aṣābahum*”
“and those who are patient over whatever has afflicted them” (Quran 22:35)

32. 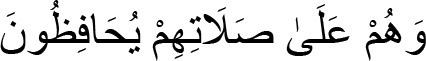
“*wahum* ^
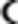
^*alā ṣalātihim yuḥāfiẓūna*”
“and they are guarding over their prayers” (Quran 6:92)

33. 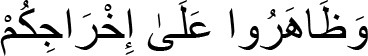
“*waẓāharu* ^
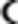
^*alā ikh'rājikum*”
“and aid in your expulsion” (Quran 60:9)

In traditional linguistics, the use of “*alā*” with acts and events cannot be explained, as acts and events are not literally physical things or space. This usage can be explained within the framework of CMT, as CMT holds that acts and events are mapped as physical objects (Lakoff and Johnson, 1980). The acts “*mā fa* ^
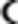
^*altum*-what you have done” in (30), “*mā aṣābahum*-whatever has afflicted them” in (31) map the deeds and acts as things, while, “*ṣalātihim*-their prayers” in (32) and “*ikh”rājikum*-your expulsion” in (33) map the acts and events as objects, as if they were possessions. The spatial preposition “^
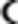
^*alā*” is used to denote the acts and events in physical terms. Moreover, contextually, “^
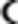
^*alā*” gets the meaning of proximity and examining in (30) and (31) because LM (acts-things) has direct influence on TR (people). In (32) and (33), “^
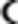
^*alā*” carries the sense of proximity and control because TR (people) has control over the LM (act and activity), respectively.

### States Are Location

The analysis revealed six examples of the STATES ARE LOCATIONS metaphor in the corpus. The analysis below illustrates this metaphor.

34. 
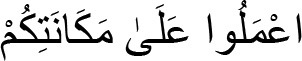
“*i* ^
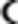
^'*malū* ^
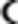
^*alā makānatikum*”
“work according to your position” (Quran 11:93)

35. 
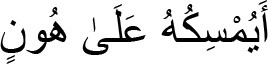
“*ayum'sikuhu* ^
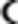
^*alā hūnin*”
“should he keep it in humiliation” (Quran 16:59)

36. 
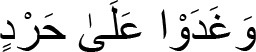
“*waghadaw* ^
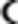
^*alā ḥardin*”
“and they went early in determination” (Quran 68:25)

In the above examples, states are conceptualized as spaces or locations. In (38) the space occupied by people is used to map their power in doing something. However, in (39) and (40), the abstract concepts of “*hūnin*-humiliation” and “*ḥardin*-determination” are given spatial configuration through “^
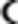
^*alā*”. The states of “position”, “humiliation”, and “determination” are Landmarks that support the TRs of “people”, the pronouns “he” and “they”, respectively.

### Personification

Supernatural entities and phenomena are treated as physical beings, and forty-two examples of this metaphor were found in the corpus. A personification is a metaphor, and, in cognitive linguistics, it is related to the Great chain of being metaphor. Lovejoy (1963) elaborated philosophically the concept of the great chain of being and tried to explain it as that the Universe consisted of plentitude, continuity, and gradation in a hierarchical fashion. The same term, the great chain of being, is at work both in poetic and common language (Lakoff and Turner, 1989). In personification, human attributes and behavior are metaphorically attributed to abstract concepts, supernatural beings, events, natural forces, complex physical or natural things, etc. The Great Chain of Being is a complex metaphorical system that employs personification in a hierarchical fashion, but not all instances of personification, in general, involve hierarchy. The following examples illustrate this phenomenon.

37. 
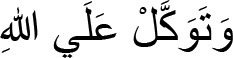
“*watawakkal* ^
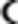
^*alā l-lahi*”
and rely upon Allah (Quran 8:61)

38. 
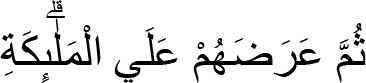
“*thumma* ^
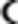
^*araḍahum* ^
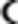
^*alā l-malāikati*”
“Then He showed them to the angels” (Quran 2:31)

39. 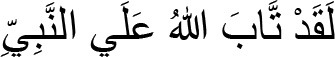
“*laqad tāba l-lahu*^
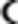
^*alā l-nabiyi*”
Verily, Allah turned in mercy to the Prophet (Quran 9:117)

The above examples show that Allah presented Himself as personified Being through language to allow humans to understand the message of the Quran. The spatial preposition “^
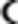
^*alā*” gives a physical connotation to the Supernatural Being, and if the unseen realm and description of supernatural entities were not described in mundane terms, humans would not understand the message (Berrada, 2006). In (37), Allah is personified upon which one can rely on, and reliance is mapped as physical support on someone”s physical strength. Similarly, in (38), the angels are defined in human terms through the spatial preposition “^
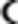
^*alā*”, where Allah, defined in human terms, showed them all the names (knowledge), as if it were something physical, and the preposition attains the semantic value of proximity, which the angels could see. In (39), Allah is personified, Who can turn toward someone, and “^
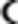
^*alā*” reflects proximity between Allah and His messenger. In (38) and (39), there is a sense of proximity, but, in (38), the context reveals that the things are in front of the angels, while (39), Allah is above His messenger, yet near to him. These implications cannot be explained in traditional theories of grammar, but cognitive linguistic approach offers plausible explanation for use of “^
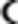
^*alā*” with abstract supernatural entities. Moreover, (38) shows CMs of the Great chain of being, and ideas are objects.

### Life Is a Journey

The concept of journey maps pervasively the concept of life. Forty-one instances of this metaphor were found in the corpus; three of which are analyzed below.

40. 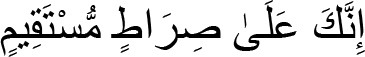
“*innaka* ^
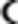
^*alā ṣirāṭin mus'taqīmin*”
“Indeed, you are on the path straight” (Quran 43:43)

41. 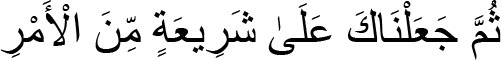
“*thumma ja*^
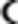
^*alnāka* ^
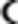
^*alā sharī*^
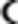
^*atin mina l-amri*”
“Then, We put you on an ordained way concerning the matter” (Quran 45:18).

42. 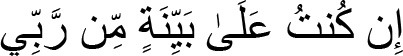
“*in kuntu* ^
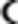
^*alā bayyinatin min rabbi*”
“if I should be upon clear signs from my Lord” (Quran 11:28)

In the Quran, LIFE IS A JOURNEY is consistently used to describe life as a spiritual and moral journey in spatial terms (see Shokr, 2006). In the above examples, the nouns “*ṣirāṭin*–path”, “*shari*^
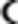
^*atin*-an ordained way” and “*bayyinatin*-clear signs” are spatial terms derived from the gestalts of journeys to map the spiritual life of human beings. The preposition “^
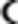
^*alā*” is used before all the nouns to give a spatial sense of support to subject pronouns. However, this sense of support is metaphorical, keeping in view the abstract nature of a spiritual journey.

### Mind Is Heart/Heart Is Container

The analysis revealed 24 examples of the CM Heart is container, but contrary to the preposition “*fi*”-”*in*”, the noun is preceded by the preposition “^
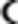
^*alā*”. This finding is more important as the preposition of containment is usually “*fi*”-“*in*” both in Arabic and English. Analysis of the following examples revealed the CM (HEART IS CONTAINER).

43. 
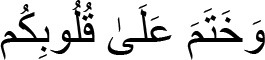
“*wakhatama* ^
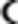
^*alā qūlubikum*”
“and set a seal upon your hearts” (Quran 6:46)

44. 
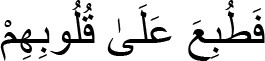
“*faṭubi*^
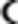
^*a* ^
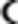
^*alā qulūbihim*”
“so their hearts were sealed over” (Quran 63:3)

45. 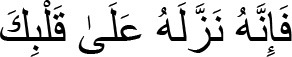
“*fa-innahu nazzalahu* ^
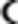
^*alā qalbika*”
“it is he who has brought the Qur”an down upon your heart” (Quran 2:97)

In these examples, “^
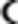
^*alā*” is metaphorically used with the sense of on or upon, because, literally, seal cannot be put on the heart. In metaphorical sense, the heart is conceived as a container that is having some opening where something can enter it and that it can be sealed. In fact, in most cultures, heart-mind metonymy is used where the heart maps the mind. The heart is conceived as the seat of thinking and planning and emotions, which is also represented as a container in language (Maalej, 2008; Sardaraz and Ali, 2017). In (43) and (44), “^
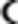
^*alā*” connotes the sense of contact metaphorically, implying that their hearts cannot understand the truth of the message of Islam. But, in (45), the sense is changed, and it takes the sense from above into the heart. The revelation is represented as if it was sent down from above and entered the heart, implying that the Book was revealed upon the Prophet, and he understood it in letter and spirit. The preposition “^
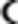
^*alā*” in these instances gives the sense of verticality and contact.

## Discussion

The analysis reveals that the preposition “^
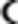
^*alā*” is extensively used in spatial terms in the Quran, which supports the traditional Arabic linguists' stance, regarding it as a locative preposition or a place relator, with the primary sense of “*isthe”lā*”-“*elevation*”, and which can be used in many other senses (Al-Sayyuti, 1982; Andulusi, 2008; Badawi et al., 2013). In addition, this view also supports the cognitive linguists' view that our experiences with outside physical world contribute to our understanding of the abstract terms we come across in our daily lives. Cognitive linguists hold that the human experiential gestalt of viewing things in the physical world helps them to structure abstract terms as objects in the physical world (Johnson, 1987; Lakoff and Johnson, 1999; Dodge and Lakof, 2005). Thus, both the traditional and cognitive linguist views of the preposition “^
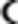
^*alā*” converge on its usage in the strict geometrical sense of locating a physical object in space with the meaning of elevation and contact. However, they diverge regarding the use of preposition “^
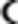
^*alā*” with abstract terms, emotional states, action, events, and supernatural entities. The former simply claims that “^
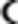
^*alā*” has several meanings besides its own basic meaning of spatial particle, while the latter views the phenomena from purely scientific standpoint, as grounded in the human sensory-perceptual system.

This paper supports the tenets of CMT that the support schema underlies the spatial preposition “^
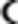
^*alā*”. Our perception of the geometrical relationship between physical objects in relation to each other and in relation to space, retrieved through our sensorimotor neural system and stored in our conceptual system, is projected to metaphorically structure the relationship of human beings to the various abstract concepts, emotional states, and supernatural phenomena, as the analysis suggests. The findings offer support for the cognitive linguistic view of grammar as a neural system rather than an abstract system (Lakoff and Johnson, 1999), and that the preposition “^
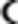
^*alā*” in language is the result of the support schema in our conceptual system. This paper supports the earlier findings concerning the spatial preposition “^
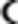
^*alā*” by Jan (2018) and English on by Ferrando (1999) insofar as “^
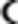
^*alā*” appears to be generated by the support schemata, and that the primary sense of the preposition is that the trajector (see Figure 1) is located higher than the landmark but is supported by landmark as the two have actual contact. However, where Jan (2018) elaborated the semantic complexity of “^
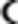
^*alā*” in its spatial usage, this study extends that its primary meaning is projected onto its usage in metaphorical senses.

Previous literature has mainly focused on the polysemous nature of the preposition “^
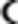
^*alā*” from a cognitive linguistic approach (see Jan, 2018), while this paper focuses on the CM approach to the semantics of “^
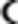
^*alā*”. The analysis reveals that various CMs, such as duties are burden, up-schema, abstract concepts/events, and acts are objects; states are locations; life is journey; Great Chain of Being metaphor and the heart is container metaphors, illustrate the view that experiential structures in our sensory-motor neural system map abstraction - abstract concepts, emotional states and events, acts, and supernatural entities in spatial and physical terms. If these abstractions had not been defined in our physical experiential concepts, the meaning of “^
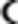
^*alā*” in spatial terms with abstract terms would not be justified. In other words, mapping in our conceptual system justifies the spatial meaning of “^
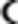
^*alā*” with abstract concepts.

Moreover, one of the important findings is that while, in English, the prepositions *above* and *over* are generally used to show graded importance between two things, as is the case with *he is above me in the company* and *he preferred me over my brother*, in Arabic, the preposition “^
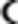
^*alā*” is used to show the importance of one thing over another. The three English translations of the preposition “^
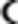
^*alā*” are “over” or “above” in the following.

46. 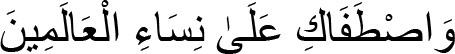
“*wa-iṣ'ṭafāki* ^
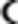
^*alā nisāi l-*^
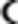
^*ālamina*”
“and chosen you above the women of the worlds” (Sahih International)
“and hath preferred thee above (all) the women of creation.” (Pickthall)
“and purified thee- chosen thee above the women of all nations.” (Quran 3:42)
(Yousaf Ali)

47. 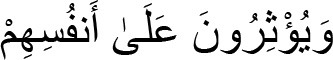
“*wayu'thiruna* ^
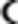
^*alā anfusihim*”
“but give [them] preference over themselves” (Sahih International)
“but prefer (the fugitives) above themselves” (Pickthall)
“but give them preference over themselves” (Quran 2:253) (Yousaf Ali)

The above examples reflect that Ali and Sahih International's translation of “^
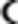
^*alā*” as *above* and *over* in both the verses, respectively, are according to the proto-sense of English prepositions. The proto-sense of *above* is verticality without the necessary influence of LM on the TR, but, in case of *over*, TR should be within the sphere of LM's influence. In (46), TR (Maryam) does not have direct influence on LM (women of the world), but, in (47), TR has impact on the LM (themselves) because it connotes that Muslims leave something for the sake of another. This shows that, whereas, in English, different particles are used for different senses, Arabic conflates in “^
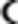
^*alā*” the spatial particles of above, on/upon, and over. This shows that cross-linguistic investigation of semantic variations and similarities of the same preposition would further contribute to cross-linguistic and cross-cultural debate on different senses of a particular preposition and CMT. The Power is up metaphor that exhibits the same difference between English and Arabic, as in the following.

48. 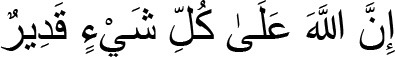
“*inna l-laha* ^
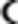
^*alā kulli shayin qadīrun*”
“Allah is over all things competent.” (Quran 2:20)

49. 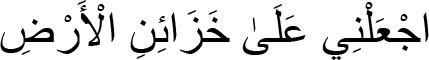
“*ij'* ^
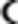
^*alni* ^
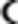
^*alā khazāini l-arḍi*”
“Appoint me over the storehouses of the land”(Quran 12:55)

In (48) and (49) both exhibit the sense of proximity and control, but Arabic use “^
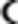
^*alā*”, whose proto scene is support, English uses “*over*” for proximity and control, whose proto scene is “*higher than*” (Tyler and Evans, 2001).

While the previous literature on spatial preposition “^
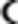
^*alā*” focused more on its polysemy (Jan, 2018), this paper focused on the metaphors used to conceptualize the non-spatial usage of this preposition. The analysis revealed that either the preceding noun adds the metaphorical connotation to “^
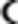
^*alā*”, or spatial nature of “^
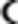
^*alā*” adds a spatial nature to abstract concepts and states. In other words, “^
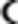
^*alā*” may be used with a spatial sense, making the following noun genitive, but, if the preceding noun is an abstract term, the whole sentence is generated as a CM, as is the case with (12), (18), (21), (26), (27), (28), (29), and (39). In cases where the following noun is a supernatural entity, the spatial nature of “^
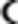
^*alā*” gives a physical character to the supernatural being as is the case in (13), (14), (17), (37), and (38). Sometimes, an abstract noun may acquire a spatial nature when the spatial preposition “^
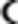
^*alā*” comes after it and so have a metaphorical nature as is the case with (18), (24), (25), (26), (32), (33), (35), (36), (41), and (42). In other cases, such as (30) to (33), an act or an event may acquire a spatial configuration through the use of “^
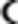
^*alā*”, while, in some cases, a spatial source domain may be used to give a spatial characteristic to the target domain through “^
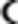
^*alā*” as is the case in (34), (40), (41), and (43) to (45). These show that, although “^
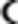
^*alā*” may be used in different contexts and in different constructions, they all suggest the basic schema of contact and support, either in the actual configuration of TR within the LM or in implied configuration of the TR within LM. Thus, the main schema is the same for all cases, but sub-schemas may give rise to different structures within the human conceptual system. According to Lakoff and Johnson (1980), human perceptual system helps in unconscious configuration of an object within the space, with reference to another object, and it generates the spatial prepositions. However, the analysis also found that language in its contextual use shows extensive semantic complexity, which requires a more elaborate mechanism for language comprehension, as discussed above, and advocated in the literature (Sardaraz and Ali, 2017, 2019, 2021). Besides the basic sense of support or contact, “^
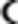
^*alā*” also reflects senses of covering and occlusion, proximity, control, elevation or higher than, more, superiority, and examining. These senses point to the fact that CM may structure the concepts, yet a preposition takes its semantic value from the context (Evans, 2010; Sardaraz and Ali, 2017).

The analysis also suggests that a single utterance may involve more than one CM as was found in (10), (17), (18), (27), (29), and (38) and that metaphor comprehension involves an integrated mechanism of different perceptual schemata and languages. In other words, metaphor comprehension may involve more than one CM and linguistic context. Similar findings have also been reported in previous literature (see Evans, 2013; Sardaraz and Ali, 2019, 2021). Therefore, this paper suggests further research into spatial relationships across languages to explore the structure of the mental lexicon and CMs in diverse cross-cultural contexts.

## Conclusion

The analysis revealed diverse CMs to structure abstract concepts, events, acts, and events in spatial terms. Different CMs underpinned the use of these non-spatial relationships structured in terms of spatial relations, but all the structures emanate from the core schema of contact and support. However, the analysis suggests that contact and support may be actual or implied, and/or there may be contact but no support, or there may be support but no actual contact. This implies that language can be used to represent a wide breadth of spatial relations, which may vary from context to context. Moreover, Arabic conflates in “^
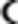
^*alā*” the English prepositions *on, over*, and *above*, although it has also separate preposition for *above*-“*fawqa*”. The sensorimotor neural system may be behind the generation of spatial prepositions, but CMT cannot account for polysemy nor can it account for any a plausible framework for the multimodal processing of perceptual schemata in language production and comprehension. Moreover, the principled polysemy model would far better contribute to the understanding of prepositions if it is combined with CMT. This paper suggests further studies on spatial prepositions to further investigate the cognitive linguistic view of spatial relations in grammar in cross-cultural contexts.

## Data Availability Statement

The original contributions presented in the study are included in the article/supplementary material, further inquiries can be directed to the corresponding author/s.

## Author Contributions

KS proposed the idea of the article and writing the first draft. RR designed the methodology of the article and worked in the analysis of the data. AN helped in the first collecting the data, analysis, and reviewed the first draft. All authors contributed to the article and approved the submitted version.

## Conflict of Interest

The authors declare that the research was conducted in the absence of any commercial or financial relationships that could be construed as a potential conflict of interest.

## Publisher's Note

All claims expressed in this article are solely those of the authors and do not necessarily represent those of their affiliated organizations, or those of the publisher, the editors and the reviewers. Any product that may be evaluated in this article, or claim that may be made by its manufacturer, is not guaranteed or endorsed by the publisher.
